# A pilot study of viscoelastic agent to prevent recurrent vitreous hemorrhage after vitrectomy for proliferative diabetic retinopathy

**DOI:** 10.1186/s12886-022-02666-7

**Published:** 2022-12-22

**Authors:** Chang-Yu Qiu, Yuan-Yuan Shi, Hong-Wei Zhao, Yu-Bo Gong, Chuang Nie, Meng-Ge Wang, Rui Jia, Jun Zhao, Xin Wang, Ling Luo

**Affiliations:** Department of Ophthalmology, Strategic Support Force Medical Center, Anxiang Bei 9#, Beijing, China

**Keywords:** Proliferative diabetic retinopathy, Vitreous hemorrhage, Vitrectomy, Recurrent vitreous hemorrhage, Viscoelastic agent, Triamcinolone acetonide, Incidence

## Abstract

**Background:**

To evaluate the possibilty of preventing recurrent vitreous hemorrhage (RVH) after vitrectomy in proliferative diabetic retinopathy (PDR) patients with unabsorbed vitreous hemorrhage (VH) by intravitreal injection of viscoelastic agent (VA) at the end of the surgery and compared its effect with triamcinolone acetonide (TA).

**Methods:**

This was a pilot prospective, observational study. PDR patients with VH who underwent vitrectomy were assigned to 3 groups according to the tamponade applicated at the end of the surgery, including VA group (intravitreally injected 1 ml VA if the retina was prone to bleed during the operation), TA group (intravitreally injected 2 mg TA when there was much exudates), or balanced salt solution (BSS) group (no tamponade). Then postoperative follow-up was performed routinely until 6 months after surgery. The primary outcome was the incidence of RVH, secondary outcome were the best-corrected visual acuity (BCVA) and introcular pressure (IOP). Cataract formation and other complication were also assessed.

**Results:**

A total of 68 eyes, from 68 patients, were included. 18,18,32 eyes were enrolled in the VA group, TA group and BSS group, respectively. The integral incidence of RVH after vitrectomy was 5.6%, 5.6% and 12.5% respectively (*P* = 0.602). There was no early RVH in VA or TA group, whereas 3 early RVHs were identified in BSS group, however there was no significant difference (*P* = 0.171). Every group had one late RVH case. In all groups, final BCVA showed significant improvement compared to baseline. BCVA at any postoperative visit showed no significant differences among 3 groups. Mean IOP was higher 1 week after surgery in VA group compared with the other groups; however, in other times the differences were not significant. No cataract formation and other complication was noted in 3 groups.

**Conclusion:**

Intravitreal injection of VA or TA at the end of vitrectomy for PDR patients with unabsorbed VH tend to reduce the incidence of early RVH after vitrectomy similarly. As VA was preferred to applicate in the eyes that were prone to bleed, intravitreal injection of VA at the end of vitrectomy might be a promising method for preventing RVH in PDR patients.

## Background

Proliferative diabetic retinopathy (PDR) is one of the leading causes of blindness in the working population [1]. PDR is characterized by retinal ischemia and neovascularization, which leads to vitreous hemorrhage (VH) and tractional retinal detachment. VH is one of the most common PDR complications and the main cause for sudden vision loss in PDR patients. Pars plana vitrectomy (PPV) can be effective in the treatment of PDR by removing vitreous opacity, relieving retinal traction and performing panretinal photocoagulation (PRP). However, one of the most common postoperative complications is recurrent vitreous hemorrhage (RVH), which may cause visual impairment and require re-operation.

The incidence rate of postoperative RVH in PDR patients has been reported to range from 11.8 to 75% [2, 3]. The risk factors for RVH are complicated, including (1) systematic factors: poor glucose control or hyperglycemia, high blood pressure, diabetic nephropathy and diabetic retinopathy stage; (2) local factors: high perfusion of retinal vessels, retinal neovascularizaiton, low postoperative intraocular pressure (IOP) and high level of vitreous vascular endothelial growth factor (VEGF); (3) treatment factors: intravitreal tamponade, laser photocoagulation; (4) other factors: direct and indirect forces on eyes, such as doing strenous exercise too early after surgery. Early studies have found that the most common causes of RVH after vitrectomy are fibrovascular ingrowth at the sclerotomy sites, residual or recurrent neovascular membrane formation on the retina, and insufficient retinal photocoagulation [4–6].

There have been enormous efforts to reduce the risk of RVH. The systematically antifibrinolytic administration [7, 8], and the intravitreal infusion of short-acting gas, are the common treatments to reduce the incidence of VH [9, 10], but the clinical outcome are unsatisfactory. Intravitreal anti- VEGF agents, as an adjunctive treatment before vitrectomy, for complicated PDR has been advocated. There were some studies evaluating the effects of anti-VEGF agents application at the end of surgery for reducing RVH incidence in patients with PDR. However, its role on preventing RVH has not been consistent [11]. Silicone oil was reported to decrease RVH incidence [12]. Kharrat et al. [13] reported that the use of silicone oil in vitrectomy for complicated PDR contributes a hemostatic and plugging effect, but it still has a number of disadvantages such as the need to remove it and its own side effects. We noticed that viscoelastic agent (VA) which is also transparent, gelatinous and commonly used to support anterior chamber space, might also has the hemostatic effect because of the similar physicochemical property to silicone oil. Besides, it could hydrate spontaneously with no need to remove it through another surgery. And before it hydrate, VA would occupy the space and help to maintain the postoperative IOP, and thus avoid postoperative low IOP which was one of the RVH risk factors [4]. We supposed that VA was a potential tamponade to prevent RVH. Besides, early studies indicated that triamcinolone acetonide (TA) had potential roles on preventing RVH [14, 15] after PPV and intraocular proliferation [16]. We conducted this pilot prospective, observational study to observe the RVH rate, vision improvement, IOP and the side effects to see whether application of VA or TA at the end of vitrectomy for PDR with non-clearing VH is helpful for decreasing the rate of RVH, and compared between them to see which way is better.

## Methods

### Ethical approval

The current study was conducted on the basis of Declaration of Helsinki principles and was approved by institutional review board of The Strategic Support Force Medical Center. Written informed consent was obtained from all participants.

### Methods

The study was designed as a prospective, observational case series. From Jan 7th in 2019, to Oct 19th in 2021, PDR patients with non-clearing VH who underwent successful micro-incision vitrectomy surgery in our department were included.

VH cases included not only simple non-clearing VH cases, but also the cases with fibrovascular proliferation, and macula involving or macula-threatening tractional retinal detachment. We excluded cases with a history of previous PPV or intravitreal anti-VEGF within the 3 previous months, other coexisting ocular disorders such as glaucoma or uveitis, silicone oil or inert gas injection during the operation, coagulopathy, receiving anti-coagulation medicine or renal failure requiring long-term dialysis.

A variety of pre-, intra-, and postoperative patient characteristics were collected, including patient age, gender, best corrected visual acuity (BCVA), intraocular pressure (IOP), any subsequent visits records including fundus images. Preoperative tests such as fasting blood sugar levels (FBSL), systolic blood pressure (SBP), serum creatinine (Cr), blood urea, prothrombin time (PT), and prothrombin standardisation ratio (PTINR) were also conducted to exclude surgery contraindications.

All patients received pre-anti-VEGF therapy 3–5 days before vitrectomy. All patients underwent 23-gauge transconjunctival vitrectomy (performed by Dr. CY Qiu who was a subspecialist vitreoretinal consultants and had more than 20 years of experience) under retrobulbar anesthesia. First, the trocar and infusion cannula were inserted. Then 3.0 mm phacoemulsification surgery was performed to remove the clouded lens as needed, and an intraocular len was implanted. And then 23-gauge transconjunctival vitrectomy was performed using the Alcon Constellation vitrectomy system (Ft Worth, TX, USA). Total vitrectomy was performed in every case; peripheral vitrectomy was completed with scleral indentation under a wide-angle visualization system. TA was used to ensure that the posterior hyaloid was eliminated. Laser photocoagulation was administered to any areas of untreated retina to complete pan-retinal photocoagulation. Near the conclusion of each case, retinal breaks and residual sources of bleeding were assessed. If there was bleeding of retinal vessels, IOP would be elevated temporarily or pressing the bleeding site with the tip of the cutter directly or applying endodiathermy to stop the bleeding. At the end of surgery, after the fluid-gas exchange procedure and before closure of sclerotomy sites, with respect to the situation during the operation, different tamponade were injected into the vitreous. When there was bleeding of retinal vessels during the operation, 1 ml VA (Freda, China) would be intravitreally injected (VA Group). In detail, a whole VA was injected manually via the infusion cannula barely with resistance. Because VA was heavier than the perfusion fluid, no VA would be expelled via the other cannulae; When there were a lot of hard exudates in the macular area, 2 mg TA would be intravitreally injected (TA group); The other cases received no tamponade but only balanced salt solution (BSS) which was the perfusion fluid commonly used during the vitrectomy (BSS group). Sutures were placed at leaking sclerotomy sites in order to avoid postoperative hypotony. At last, TobreDex eye oint were placed on the surface of the eye followed by a patch. No silicon oil or inert gas was used.

Patients were followed-up routinely at 1 day, 1 week, 2 weeks, 4 weeks, 2 months, 3 months, and 6 months post-operation. RVH was considered if there was vitreous opacity which caused retina obscure and assessed by three vitreoretinal subspecialists. Meanwhile, BCVA and IOP was also examined. The onsets of cataract formation, retinal detachment, endophthalmitis and other complications were assessed through the follow-up period.

### Statistical analyses

All analyses were performed using SPSS version 21.0 for Windows. Descriptive statistics were determined. To compare ratio or incidence rate between groups use Chi-squared test. Average value or mean distribution were determined using T-test or ANOVA. Binomial logistic regression analyses and multivariate analysis were also performed. For all statistical tests, *P* < 0.05 was considered significant.

## Results

**Fig. 1:**
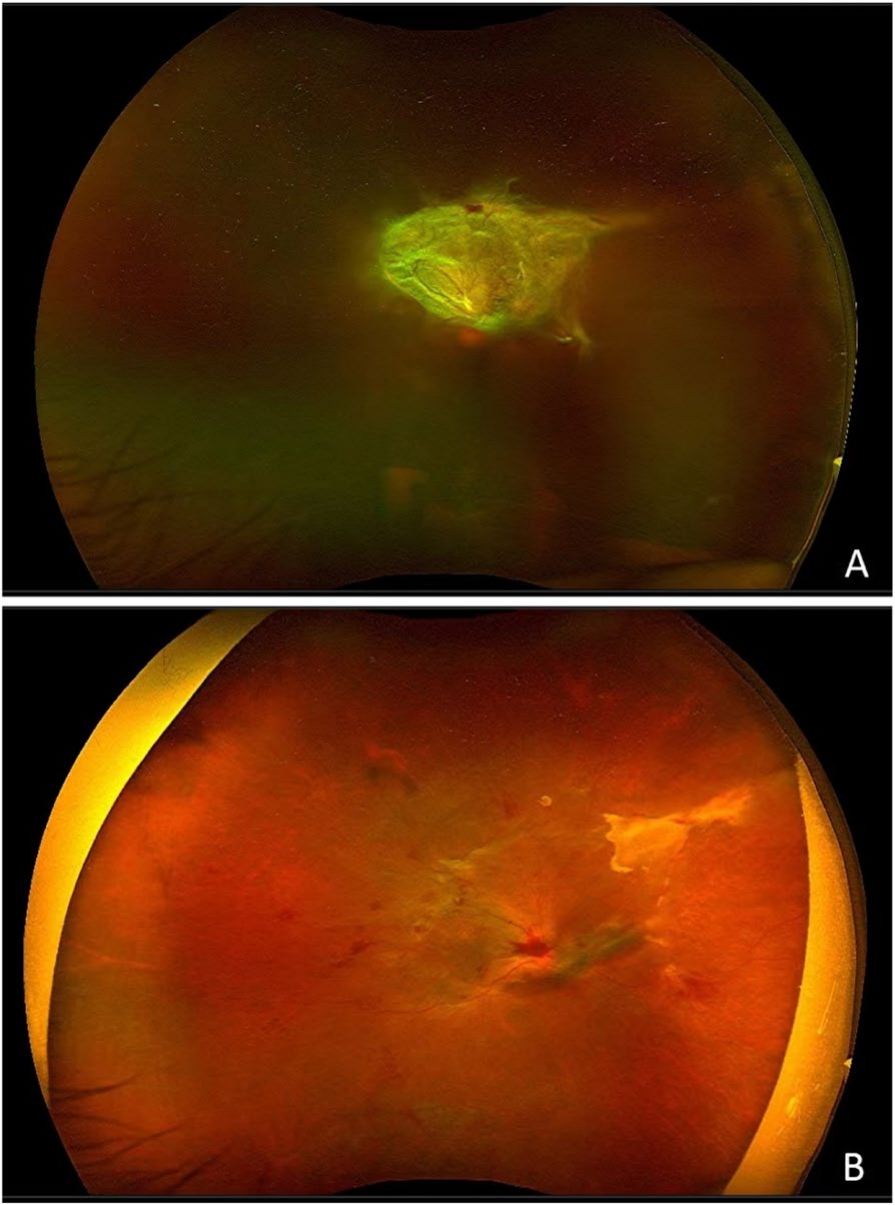
Fundus images of one case in VA group before and after surgery. **A** Preoperative fundus image showed unclear fundus due to the vitreous hemorrhage and a lot of fibrovascular membrane above the optic disc. **B** Fundus image 1 week after surgery showed clear fundus and a little hemorrhage. Viscoelastic agent in the vitreous cavity was invisible and showed no reflections

Finally, a total of 68 eyes, from 68 PDR patients with non-clearing VH who met the inclusion criteria and had completed 6 months of follow-up were included in this study (18 eyes in VA group, 18 eyes in TA group, 32 eyes in BSS group respectively). There were 27 women and 41 men with a mean age of 57.8 ± 9.3 years. As detailed in Table 1, there were no statistically significant differences in terms of age or gender when compared among 3 groups. 8 eyes (44.44%) in VA group, 11 eyes (61.11%) in TA group and 15 eyes (46.88%) in BSS group received combined phacoemulsification surgery. During the follow-up period, totally 6 eyes were recognized as RVH and the global incidence of RVH was 8.82%. All of the RVH was graded 4 according to the grading system for VH. RVH occurred in 1 (5.56%), 1(5.56%) and 4 (12.5%) cases of VA group, TA group and BSS group respectively, as shown in Table 1. There were no significant difference between 3 groups (χ^2^ = 0.957, *P* = 0.602). The RVH case in VA group experienced RVH at 3 months after surgery and reoperation of vitrectomy was carried out. The RVH case in TA group experienced RVH at 1 month and VH was absorbed spontaneously. RVH in BSS group occurred at various time including 3 early RVH cases (2 cases at 1 day and 1 case at 3wk) and 1 case at 3 months after surgery. Reoperation of vitrectomy was carried out in 2 cases of the BSS group, and in the other 2 cases, VH was absorbed spontaneously. The incidence of early RVH showed no significant difference (*P* = 0.171). VA in the vitreous cavity were invisible and did not show any opacity or reflections during the follow-up. Pre- and postoperative fundus images of one case in VA group were shown in Fig. 1.

**Table 1:** The occurrence of recurrent vitreous hemorrhage and patient data before and after surgery

	VA group	TA group	BSS group	*P* value
Mean age,(yrs)	56.9 ± 11.0	58.1 ± 8.7	58.8 ± 9.9	0.921
Gender,(male/female)	12/6	10/8	19/13	0.784
Cases(n)	18	18	32	
Cases combinedly received phacomulsification surgery (n)	8	11	15	
RVH(n)	1	1	4	
early RVH(n)	0	0	3	
Late RVH(n)	1	1	1	
Incidence of RVH(%)	5.56	5.56	12.5	0.602
Incidence of early RVH(%)	0	0	9.4	0.171

In all groups, BCVA dramatically improved postoperatively as illustrated in Table 2. However, no significant difference in BCVA was found between the groups (*P* = 0.989 for baseline, *P* = 0.801 for 1wk and *P* = 0.481 for 6 months). IOP increased only in VA group at 1wk after surgery, as illustrated in Table 3. In the VA group, IOP increased significantly from (15.83 ± 2.36) mmHg at baseline to (21.67 ± 10.39) mmHg at 1wk (*P* = 0.028) and then decreased to (16.22 ± 2.86) mmHg at 6 months (*P* = 0.170). There was no significant difference at at any time points in the TA and BSS groups. When compared among groups at the same time, IOP at 1wk in VA group were significant higher (*P* = 0.026). Patients whose IOP were high at 1wk received local ocular hypotensive eyedrops or paracentesis of anterior chamber. The IOP was mostly normal at 2wk. Little reaches normal at 3 wk.

**Table 2:** BCVA at different time points of three groups

	Baseline BCVA (logMAR)	BCVA at 1wk (logMAR)	BCVA at 6 months (logMAR)
VA group	1.37 ± 0.52	0.73 ± 0.39^a^	0.46 ± 0.37^a^
TA group	1.38 ± 0.46	0.74 ± 0.37^a^	0.71 ± 0.56^a^
BSS group	1.31 ± 0.49	0.83 ± 0.52^a^	0.58 ± 0.39^a^

^a^ compared with baseline, *P* < 0.05

**Table 3:** IOP at different time points and groups

	Baseline IOP (mmHg)	IOP at 1wk (mmHg)	IOP at 6 months (mmHg)
VA group	15.83 ± 2.36	21.67 ± 10.39^ab^	16.22 ± 2.86
TA group	16.44 ± 1.50	18.14 ± 9.50	16.57 ± 2.24
BSS group	15.81 ± 1.86	16.22 ± 6.22	16.44 ± 2.17

^a^ compared with baseline, *P* < 0.05; b:compared with TA and BSS group at 1wk, *P* < 0.05

A binomial logistic regression analysis revealed no risk factors of RVH. Age, gender, tamponade at the end of the surgery, vision improvement and IOP change did not differ significantly between cases with or without RVH in 3 Groups.

The onsets of retinal detachment, endophthalmitis, cataract formation or any other ocular complications were not observed in all of the groups through the observational periods.

## Discussion

The purpose of this study was to evaluate possible treatments to decrease the probability of RVH in patients who received PPV for nonabsorbent VH due to PDR. In this study, we intravitreally injected VA and TA at the end of the surgery. Although not statistically significant, the incidence in VA and TA group was lower than BSS group, especially the incidence of early RVH.

The causes of RVH are different according to the VH time. Early RVH was defined as VH occurring within 1 month after surgery while late RVH was defined as VH occurring between 4 weeks and 12 months after surgery. As we know, the causes of early RVH appeared to be dispersion of remnant blood clots from the vitreous base, bleeding from remnant fibrovascular tissues spontaneously or after coagula attached to the vessels fell off [3, 17, 18]. The etiology of late RVH is considered to involve sclerotomy sites neovascularization, along with anterior hyaloidal fibrovascular proliferation [11, 19, 20].

In this study, for the first time, we intravitreally injected 1ml VA at the end of surgery and we prefer to use it when there was bleeding of retinal vessels in fibrovascular tissue which was controlled by temporarily raising the intraocular pressure and pressing the bleeding site with the tip of the cutter directly, or applying endodiathermy during the operation. That is to say we tend to use VA in the eyes that prone to bleed. However, of 18 eyes in VA group, there was only one severe RVH case at 3 month after surgery and especially no early RVH was identified. The integral incidence was 5.6%, lower than BSS group and also some previous results [2, 3, 11]. It implied that VA was to a extent prevent RVH especially early RVH. As we all know, VA was commonly and safely used in ocular surgery. It is viscous and can occupy the space which somewhat likes the silicon oil. The characteristic may help to reduce the fluidity of vitreous humor and reduce the dispersion of remaining blood. It might also buffer the shear or scouring force of vitreous liquid on retina vessels when there was eye movement and the relative motion of vitreous liquid and retina would produce. And thus VA might reduce the risk of rebleeding of vascular stumps and falling off of coagula attached to the vessels. Meanwhile the VA would take several days to hydrate completely or being discharged. During this time, the IOP would be higher than normal as the VA was discharged through the trabecular meshwork and somewhat block the passage. Besides, VA occupied the vitreous cavity, but aqueous humor constantly produced, so the volume would beyond the normal and thus resulted in high IOP. Early postoperative hypotony after PPV has been suspected to be associated with an increment of RVH [4, 21]. Lee et al. [3] had showed that patients who experienced postoperative hypotony had an 11.20-fold increased risk of immediate RVH. Soto-Pedre et al. [22] suggested early peaks of RVH occurs at the end of the first week. So, to the contrary, higher IOP in the early stage after PPV may be a protect factor. In this study, the IOP averaged at 21.67±10.39 at 1wk in VA group. The IOP elevation of VA group mostly began at 3 or 4 day and continued during the first week after surgery. And most of the IOP decreased to normal in the 2nd week under ocular hypotensive treatment. Although VA kept invisible, the elevated postoperatvie IOP implied that VA probably persisted in the vitreous cavity between one to two weeks. So we believed that VA probably could prevent immediate or early RVH.

To compare the role of VA with other existing tamponade, we also intravitreally injected 2 mg TA at the end of the surgery. There was only one RVH case at 1 month after surgery, and the VH was absorbed spontaneously. TA is a water-insoluble and long-acting steroid hormone that has been commonly and safely used to assist vitrectomy for PDR, anti-inflammation or macular edema in eyes for many years [16, 23, 24]. During the study, we prefer to use TA in the cases that had prominent macular edema or much exudation during the operation. Previously, intravitreal injection of TA in PPV surgery at different time for PDR patients has been evaluated for preventing RVH, but the results were different. Liao et al. [15] reported that intravitreally injection of 4 mg TA after posterior vitreous detachment (PVD) during PPV for PDR could prevent RVH. The incidence of early RVH after PPV was significantly lower in the TA group (1.7%) than in the non-TA group (9.9%).They also indicated that TA injection immediately after PVD led to improved hemostasis. They found that retinal bleeding was obviously reduced or stopped after TA intravitreal injection during PPV. TA particles could cover the bleeding area and form a thin layer with red blood cells to manage intraoperative active bleeding [25]. Besides, TA was able to stabilize the blood-retinal barrier by inhibiting prostaglandins and inflammatory adhesion molecules as well as by reducing VEGF levels [26–28]. TA can maintain its effect for 2–3 weeks or even longer in the vitreous cavity as it is slowly absorbed and difficult to dissolve in liquid [29, 30]. These might explain why TA reduce the incidence of early RVH after PPV. Faghihi et al. [28] reported rate of early RVH and reoperation were significantly lower in patients taking intravitreal injection of 4 mg TA compared with other group (13.2% in TA group and 45.5% in control group); and no reoperation in TA group and 11.8% in the control, respectively. However, negative results were also reported previously. Takamura et al. [31] reported that RVH after PPV for patients with VH due to PDR occurred in 2 (4.8%) cases in TA-injection group and 3 (7.1%) cases in the non-TA injection group. The difference of the ratio was insignificant between the groups. Hu et al. [23] evaluated treatment efficacy of postoperative intravitreal 2 mg TA at the basis of preoperative intravitreal ranibizumab (IVR) in patients undergoing PPV for PDR. Patients received PPV with preoperative IVR or underwent PPV combined preoperative IVR and postoperative IV of TA. No significant difference of RVH was observed between the two groups. In our study, the overall incidence of RVH of TA group was 5.6% with no early RVH or any reoperation. The results was comparative with the previous studies. Compared with BSS group, although not significant, the incidence was lower, especially the early RVH. So we should not ignore that possibility that TA might help to inhibit early RVH.

In this study, the postoperative IOP did not show significant differences between the TA and BSS groups at 1 week. Actually, some patients had transient higher postoperative IOP in the TA group within the first week. However, most patients with high IOP drop to normal after ocular hypotensive treatment. This was similar to the previous studies [23, 28]. And the duration was aligned with the pharmacokinetics of triamcinolone acetonide after a single intravitreal injection. Beer et al. [32] reported that the half-life of TA in the aqueous humour was 18.6 days in the non-vitrectomised eyes, whereas it was only 3.2 days in the vitrectomised eyes.

Every group had one late RVH case. Previously, the cause of late RVH was believed to be the fibrovascular ingrowth at the sclerotomy sites [4, 33]. However, in the patients undergoing reoperation in our study, we found no neovascularization at the sclerotomy sites through scleral indentation. And we just perform simple vitreous cavity washout and retinal laser photocoagulation supplement. After the second surgery, no one had VH again during the follow up. We believed that reasons for late RVH was complicated. BCVA of three groups did not show significant difference at the end of follow-up. It seemed that there was no advantage or disadvantage in VA and TA application with respect to BCVA.

The present study had some limitations. This was a prospective, single-center study, in which all vitrectomies were performed by a single surgeon. A major limitation of this study was the presence of a selection bias because our study was not a randomized prospective study. Patients who received VA or TA injections usually had bleeding-prone lesions or inflammation indications in the fundus. BSS cases usually had relatively mild lesion based on the judgement of the surgeon. However, VA and TA group still have lower incidence of RVH within 1 month which indicates VA and TA injections had positive effects in this study. Besides, In this study we only calculated the severe RVH that we considered to have clinically significance. Nevertheless, further randomized case-controlled studies on this topic are recommended.

In conclusion, we for the first time studies the effects of IV VA on RVH after vitrectomy for PDR and found the overall incidence of RVH in VA group was lower than in BSS group, especially the early incidence of RVH. Although the integral incidence is not statistically significant, we could not ignore the trend. Besides, we evaluated the role of TA simultaneously, and VA seemed to have a comparative role with TA, whereas may avoid the side effects of TA such as promoting cataract formation. The temporary increased IOP in VA group might be one of the effective cause or a disadvantage which should be assessed further. In all, VA may be a potential tamponade agent that can be used to prevent RVH in PDR patients especially the ones that prone to bleed during the operation. Randomized case-controlled studies with a larger number of cases would be required to confirm our findings.

## Data Availability

The datasets used and/or analyzed during the current study are available from the corresponding author on reasonable request.

## Abbreviations

OCT: Optical coherence tomography
BCVA: Best corrected visual acuity
MIVS: Micro-incision vitrectomy surgery
LogMAR: Logarithm of the minimum angle of resolution
VA: Viscoelastic agent
TA: Triamcinolone acetonide
IV: Intravitreal injection
BSS: Balanced salt solution
RVH: Recurrent vitreous hemorrhage
PDR: Proliferative diabetic retinopathy
PVD: Posterior vitreous detachment
VEGF: Vascular endothelial growth factor
IOP: Intraocular pressure
